# Spin and Orbital Rotation of Plasmonic Dimer Driven by Circularly Polarized Light

**DOI:** 10.1186/s11671-018-2739-3

**Published:** 2018-10-12

**Authors:** Jiunn-Woei Liaw, Mao-Chang Huang, Hsueh-Yu Chao, Mao-Kuen Kuo

**Affiliations:** 1 Department of Mechanical Engineering, Chang Gung University, 259 Wen-Hwa 1st Rd., Kwei-Shan, Guishan District, Taoyuan City, 33302 Taiwan; 2 Department of Mechanical Engineering, Ming Chi University of Technology, Taishan District, New Taipei City, 24301 Taiwan; 3 Medical Physics Research Center, Institute for Radiological Research, Chang Gung University and Chang Gung Memorial Hospital, Linkou, Taiwan; 4 Center for Advanced Molecular Imaging and Translation, Chang Gung Memorial Hospital, Linkou, Taiwan; 5 Institute of Applied Mechanics, National Taiwan University, Taipei, 106 Taiwan

**Keywords:** Optical manipulation, Laser trapping, Plasmonics, Surface plasmons, Spin-orbit coupling, MMP

## Abstract

The plasmon-enhanced spin and orbital rotation of Au dimer, two optically bound nanoparticles (NPs), induced by a circularly polarized (CP) light (plane wave or Gaussian beam) were studied theoretically. Through the optomechanical performances of optical forces and torques, the longitudinal/transverse spin-orbit coupling (SOC) of twisted electromagnetic fields was investigated. The optical forces show that for the long-range interaction, there exist some stable-equilibrium orbits for rotation, where the stable-equilibrium interparticle distances are nearly the integer multiples of wavelength in medium. In addition, the optical spin torque drives each NP to spin individually. For a plane wave, the helicities of the longitudinal spin and orbital rotation of the coupled NPs are the same at the stable-equilibrium orbit, consistent with the handedness of plane wave. In contrast, for a focused Gaussian beam, the helicity of the orbital rotation of dimer could be opposite to the handedness of the incident light due to the negative optical orbital torque at the stable-equilibrium interparticle distance; additionally, the transverse spin of each NP becomes profound. These results demonstrate that the longitudinal/transverse SOC is significantly induced due to the twisted optical field. For the short-range interaction, the mutual attraction between two NPs is induced, associated with the spinning and spiral trajectory; eventually, the two NPs will collide. The borderline of the interparticle distance between the long-range and short-range interactions is approximately at a half-wavelength in medium.

## Background

The optical binding of two microparticles (MPs) or nanoparticles (NPs) irradiated by a linearly polarized (LP) light is an important optomechanical behavior, which is the result of light-matter interaction [1–4]. There are several stable-equilibrium interparticle distances between the optically bound dimer; these distances are nearly the integer multiples of wavelength in medium [3–6]. In addition, the orientation of the dimer is perpendicular to the polarization of LP light. As the interparticle distance is close to integer multiples of the wavelength, the scattered photons between particles make a constructive interference to induce a binding force. The phenomena of optically bound array of multiple silica MPs or Ag NPs were also studied [7–10]. For the illumination of a circularly polarized (CP) plane wave, Haefner et al. reported that the helicities of the longitudinal spin and orbital rotation of the two coupled silica NPs with size of 100–700 nm are the same with the handedness of the incident light [11]. Recently, Sule et al. experimentally found that the helicity of the orbital rotation of two Ag NPs of radius 75 nm bound by optical force is opposite to the handedness of a focused CP Gaussian beam of 790 nm in water [12]; i.e., Ag dimer suffers a negative optical orbital torque [13, 14]. In addition, the measured orbit rotation was about 4 kHz [12]. On the other hand, the spin of a single Au NP of radius 100 nm induced by a CP Gaussian beam has also been studied [15–18]. The measured spin rotation was as high as 3.5 kHz [15]. In recent decades, the longitudinal/transverse spin-orbit coupling (SOC) of optical field attracts a lot of attention [19–23]. For example, an optical vortex beam (e.g., high-order Laguerre-Gaussian beams with azimuthal or radial polarizations) or highly focused CP Gaussian beam can be used to induce the SOC [24–34]. The twisted electromagnetic (EM) field of the optical vortex beam carries both the spin angular momentum and orbital angular momentum, thereby inducing the longitudinal/transverse spin and orbital rotation of a nearby probing NP [18–26]. In particular, the SOC in the near field of Au or Ag NPs is more significant due to the collective motion of free electrons in these NPs (plasmon effect) [28–31].

In this paper, we theoretically study the optomechanical behaviors (optical forces and torques) of two coupled Au NPs (dimer) supported by a substrate, which are induced by the illumination of a CP Gaussian beam. The substrate is necessary to confine these freestanding NPs moving in the focal plane, instead of floating in 3D space. The multiple multipole (MMP) method is used to simulate the EM field numerically and then to analyze the optical orbital and spin torques upon the optically bound dimer [35, 36]. Through the optomechanical responses of the dimer, the longitudinal/transverse SOC will be manifested. In particular, the condition to generate a negative optical orbital torque on the dimer will also be investigated.

## Methods

**Fig. 1:**
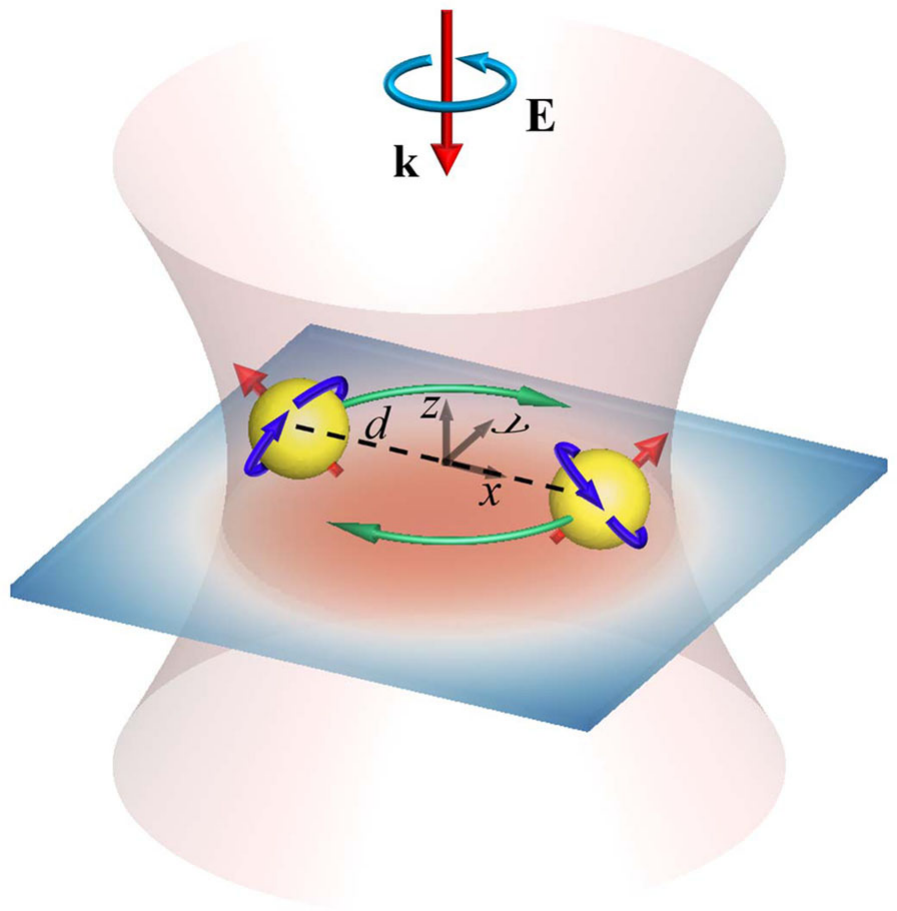
Configuration of a pair of NPs with a supporting substrate, irradiated by a normally incident LH CP Gaussian beam of waist (w0). The central cross sections of these NPs are at the focal plane of the Gaussian beam, and d is the distance between the centers of NPs. The optomechanical responses are the longitudinal orbital rotation and longitudinal/transverse spin

Figure 1 shows the configuration of a pair of identical Au NPs supported by a substrate and irradiated by a normally incident left-handed (LH) CP light (plane wave or Gaussian beam), where *d* represents the interparticle distance. The waist of the Gaussian beam is denoted by *w*_0_, and the focal plane is at the central cross section of Au NPs. The formulations of the electric field of plane wave and Gaussian beam are attached as Appendix. We assume that the refractive index of the substrate is the same as that of the surrounding medium, water. Therefore, the reflected light will not be induced at the interface between the medium and the substrate; the optical field is not disturbed by the existence of the substrate [37]. On the other hand, the existence of the substrate serves as a confinement to support NPs moving on the substrate. The multiple multipole (MMP) method is used to simulate the induced electromagnetic field [17, 18, 35, 36]. The optical forces **F**^*j*^ exerted on the *j*th NP (*j* = 1, 2) are expressed by

1 $$ {\mathbf{F}}^j={\int}_{S_j}\mathbf{T}\cdot \mathbf{n}\kern0.1em \mathrm{d}S. $$

Here, **n** is the outward normal vector on the surface of the *j*th NP, and **T** is the time-average Maxwell stress tensor expressed as

2 $$ \mathbf{T}=\frac{1}{2}\operatorname{Re}\left\{\varepsilon \mathbf{E}\overline{\mathbf{E}}+\mu \mathbf{H}\overline{\mathbf{H}}-\frac{1}{2}\left(\varepsilon \mathbf{E}\cdot \overline{\mathbf{E}}+\mu \mathbf{H}\cdot \overline{\mathbf{H}}\right)\;\mathbf{I}\right\}. $$

In Eq. (2), **I** is a 3 × 3 identity matrix, the overbar denotes the complex conjugate and Re the real part [17, 18, 35, 36]. Here, *ε* and *μ* are the permittivity and permeability of the surrounding medium. Notice that the **E** and **H** are the exterior total field used for Eq. (2). In fact, **T** is also the time-averaged linear momentum flux. Throughout this paper, the optical forces are expressed in the cylindrical coordinates: the radial, azimuthal, and *z*-axis components. The radial force can tell the attraction or repulsion between the two NPs and the azimuthal force the helicity of NPs’ orbital revolution.

On the other hand, the optical spin torque on the *j*th NP (*j* = 1, 2) for the spinning of individual NP is given by,

3 $$ {\mathbf{M}}^j={\int}_{S_j}{\mathbf{x}}^j\times \mathbf{T}\cdot \mathbf{n}\;\mathrm{d}S. $$

In Eq. (3), **x**^*j*^ × **T** is the angular momentum flux and **x**^*j*^ is the relative position vector of a point **x** on the surface *S*_*j*_ with respect to the center of mass ${\mathbf{x}}_c^j$of *j*th NP; ${\mathbf{x}}^j=\mathbf{x}-{\mathbf{x}}_c^j$. The longitudinal direction is designated to be parallel to the optical axis (say *z* direction) of the incident light, and the transverse direction is perpendicular to the optical axis. On the other hand, the longitudinal optical orbital torque in the *z* direction on each NP, caused by the azimuthal optical force, is defined as *F*_*θ*_ *d*/2 in the cylindrical coordinates. The relative permittivity of Au at *λ* = 800 nm used in the simulation is (− 24.062, 1.507) [38].

## Results and Discussion

**Fig. 2:**
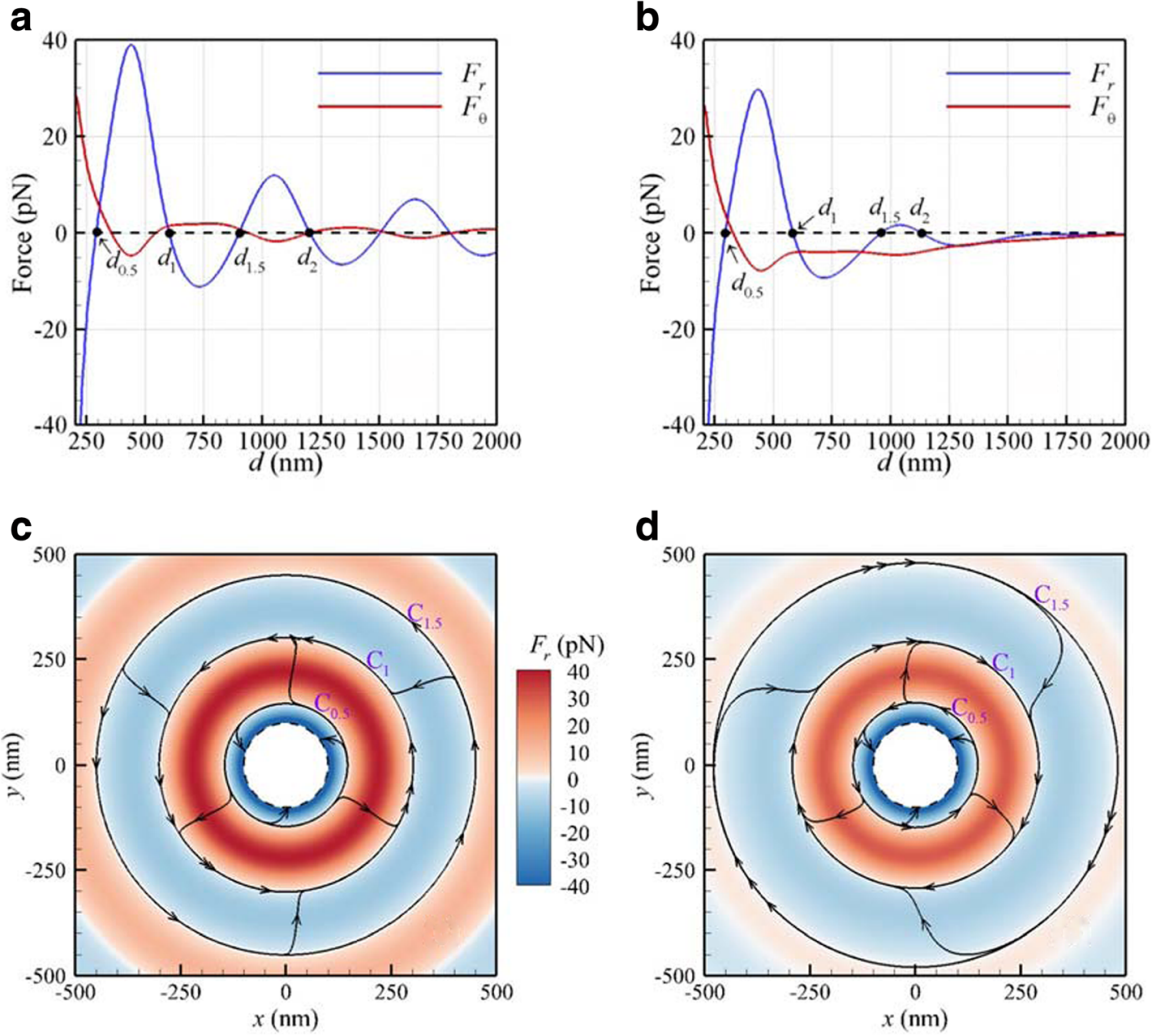
Optical forces (*F*_*r*_, *F*_*θ*_) versus *d* for *λ* = 800 nm by CP **a** plane wave and **b** Gaussian beam with a waist of 500 nm at the focal plane. The 2D streamline maps of optical force (*F*_*r*_, *F*_*θ*_) induced by CP **c** plane wave and **d** Gaussian beam. The color bar represents the amplitude of *F*_*r*_. The dashed ring is the limit circle of the centers of two NPs where NPs are in contact

We study the optical forces and torques exerted on two identical Au NPs with radii 100 nm irradiated by a normally incident LH CP plane wave or a focused Gaussian beam at the focal plane. The surrounding medium is water. The fluence of the plane wave or Gaussian beam at the center is 25 MW/cm^2^. The centers of the two freestanding NPs, supported by a virtual substrate, are allowed to move in the *xy* plane (focal plane). The optical forces (*F*_*r*_, *F*_*θ*_) versus the interparticle distance *d* for a CP plane wave or a focused Gaussian beam with a waist of 500 nm of *λ* = 800 nm are shown in Fig. 2a, b, respectively. The central cross section of these NPs is at the focal plane of the Gaussian beam. Figure 2a indicates that for a plane wave, there are several stable-equilibrium interparticle distances with *F*_*r*_ = 0 and a negative slope; the first one *d*_1_ is at 603 nm and the second one *d*_2_ at 1204 nm. These “stable-equilibrium” interparticle distances are nearly the integer multiples of wavelength in medium; i.e., *d*_*m*_=*mλ*/*n*, where *n* is the refractive index of medium and *m* = 1, 2, 3... It is a result of the long-range light-matter interaction caused by the optical binding force. It suggests that there is an optical spring connecting the two NPs; the restoring force *F*_*r*_ of the optical spring keeps NPs apart from each other at these stable-equilibrium interparticle distances. For the case of the Gaussian beam, the first two stable-equilibrium interparticle distances *d*_1_ and *d*_2_ are 585 and 1131 nm respectively, as shown in Fig. 2b, slightly smaller than those of a plane wave due to the gradient force induced by the Gaussian beam.

In fact, the longitudinal orbital torque *F*_*θ*_ *d*/2 will drive these NPs to rotate in orbits with diameters of *d*_1_ and *d*_2_. For the cases with the Gaussian beam, orbits will be centered at the beam axis. The sign of the azimuthal optical force (*F*_*θ*_) indicates that the longitudinal orbital rotation (revolution) at the first stable-equilibrium orbit induced by the Gaussian beam is opposite to that by a plane wave. This shows that the negative *F*_*θ*_ of CP Gaussian beam generates a negative orbital torque *F*_*θ*_ *d*_1_/2 at the first stable-equilibrium orbit; more importantly, the helicity of the orbital rotation of Au dimer is opposite to the handedness of the incident CP light [12]. It is also of interest to note that *F*_*θ*_ is always negative as *d* > 300 nm for the cases with the Gaussian beam; the phenomenon of reverse rotation (revolution) of the optically bound NPs, due to negative orbital torque, is easily observed in optical tweezers systems. The negative optical orbital torque could be attributed to the twisted EM field of the Gaussian beam [23].

According to Stokes’ law of a sphere driven by a force **F** to move in a viscous fluid, the terminal speed **v**_*T*_ is **v**_*T*_ = **F**/(6*πrη*), where *η* is the dynamic viscosity of water (0.001 kg/m s). This is a result of the applied force balanced by a drag force of viscous fluid [39]. Based on Stokes’ law, the terminal velocity vector of a NP in a viscous medium is proportional to the applied force [39]. Hence, we used the optical force field to obtain the streamlines, which are almost equivalent to the trajectories of these NPs. Furthermore, the 2D streamline maps obtained directly from the optical force vector field (*F*_*r*_, *F*_*θ*_) exerted on NPs are plotted in Fig. 2c, d for plane wave and Gaussian beam respectively, where the color bar represents the amplitude of *F*_*r*_. Note that the tangent of the streamline at each point is then parallel to the optical force vector and hence is also parallel to the velocity of NP. For small interparticle distance range (*d* < *d*_0.5_), the radial optical force *F*_*r*_ is negative, so these two NPs will attract each other to collide eventually, as shown in Fig. 2c, d. The dashed ring is the limit circle of the centers of two NPs where NPs are in contact. The inner annulus (blue) is a region of the short-range interaction. The inner ring *C*_0.5_ between the inner annulus (blue, with negative *F*_*r*_) and the second annulus (red, with positive *F*_*r*_) is the border line between the short-range and long-range interaction regions of Au dimer; the diameter of *C*_0.5_ is *d*_0.5_ = 291 nm in Fig. 2c and *d*_0.5_ = 296 nm in Fig. 2d. In the long range (*d*_0.5_ < *d* < *d*_1.5_), the radial and azimuthal optical forces drive the two coupled NPs to approach the first stable-equilibrium orbit *C*_1_ with a diameter *d*_1_ due to the effect of optical binding force. The optically bound Au dimer rotates counter-clockwise (CCW) along the orbit *C*_1_ (*d*_1_ = 603 nm) in Fig. 2c, whereas along *C*_1_ (*d*_1_ = 585 nm) clockwise (CW) in Fig. 2d. The former rotation is the same as the handedness of incident light caused by the positive orbital torque (*F*_*θ*_ > 0), and the latter is reverse due to the negative orbital torque (*F*_*θ*_ < 0). According to our analysis of the scattering cross section spectrum of a dimer with a stable-equilibrium distance of 603 nm irradiated by a CP plane wave (not shown here), the coupling surface plasmon resonance (SPR) of the optically bound dimer is almost at 800 nm corresponding to the incident light, which is off-resonance of a single NP (530 nm). In general, the coupling SPR of a dimer depends on the interparticle distance; the larger the distance the more red-shifted the coupling SPR of the dimer. If we use a longer-wavelength Gaussian beam (e.g., 1064 nm), the stable-equilibrium interparticle distance increases. However, as the distance between the two NPs becomes too large, the optical coupling effect decreases so the coupling SPR gradually disappears. Consequently, the SPR of a single NP at 530 nm becomes dominant.

For an Au NP of radius 100 nm which moves along an orbit with a diameter *d* and an angular speed Ω_z_, the speed is Ω_z_*d* /2 = *F*_*θ*_/(*6πrμ*). If the Gaussian beam is applied (*F*_*θ*_ = − 4 pN), the angular speed Ω_z_ (cycles per second) along *C*_1_ is about − 7 kHz. The order of magnitude is consistent with the experimental result [12]; the angular speed of the orbital rotation of two Ag NPs of *r* = 75 nm irradiated by the Gaussian beam with 14 mW is − 4 kHz. If *d*_1.5_ < *d* < *d*_2.5_, these NPs will approach and rotate along the secondary stable-equilibrium orbit *C*_2_ (not shown here). Notice that for these cases the optical force of *F*_*z*_ is negative to push these NPs downstream due to the radiation pressure; *F*_*z*_ = − 161.3 pN for plane wave and − 117.2 pN for the Gaussian beam. This infers that the reaction force from the supporting substrate is necessary to balance the driving optical force of *F*_*z*_. Consequently, the resultant forces in *z* direction upon these NPs are zero; these two NPs are confined to move in the *xy* plane of the focal plane.

**Fig. 3:**
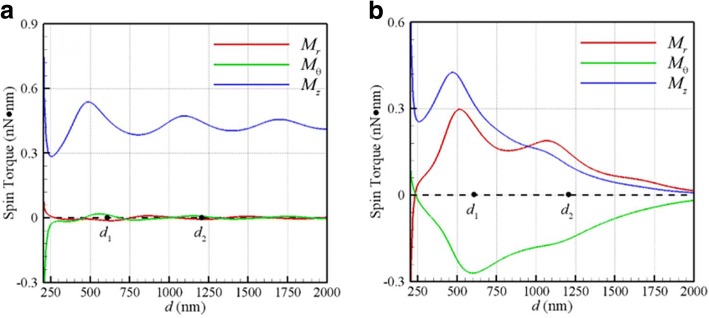
Optical spin torques (*M*_*r*_, *M*_*θ*_, *M*_*z*_) versus *d* at *λ* = 800 nm for **a** plane wave and **b** Gaussian beam with *w*_0_ = 500 nm at focal plane

On the other hand, Fig. 3a, b shows the optical spin torques (*M*_*r*_, *M*_*θ*_, *M*_*z*_) versus *d* induced by a plane wave and Gaussian beam at the focal plane, respectively. Since the results of these two NPs are the same, only a set of optical spin torques is plotted here. The former two (*M*_*r*_, *M*_*θ*_) are the transverse spin torques, and the latter *M*_*z*_ is the longitudinal one. It is found that the helicity of the longitudinal spin torque is the same as the handedness of incident light for both cases. This is because that the angular momentum of absorbed photons of incident CP light is transferred to these NPs for spinning and orbital rotation. It is of interest to point out that the transverse optical spin torques (*M*_*r*_, *M*_*θ*_) induced by a Gaussian beam are significantly large, compared to those by the plane wave. This could be attributed to the transverse components of the twisted EM field at the focal plane of a Gaussian beam. Moreover, the maximum magnitudes of the optical transverse spin torques roughly occur at the first stable-equilibrium orbit *C*_1_ (*d*_1_ = 585 nm), as shown in Fig. 3b. According to Stokes’ law of a spinning sphere rotated by a torque **M** in viscous fluid, the terminal angular velocity of the sphere is **ω**_*T*_ = **M**/(8*πr*^3^*μ*) [18]. Hence, the magnitudes of the longitudinal/transverse spin angular velocities of NP at *C*_1_ are about 10 kHz, of which the orders of magnitude are in agreement with the measured longitudinal spin velocity [15], about 3.5 kHz. Summarily, the above phenomena, as shown in Figs. 2b and 3b, demonstrate that the longitudinal orbital rotation is accompanied by the longitudinal/transverse spins. The motion of the two coupled NPs is similar to that of a binary-star system, where the optical forces provide the binding and orbital driving forces for these NPs as well as the optical spin torques cause their spinning.

**Fig. 4:**
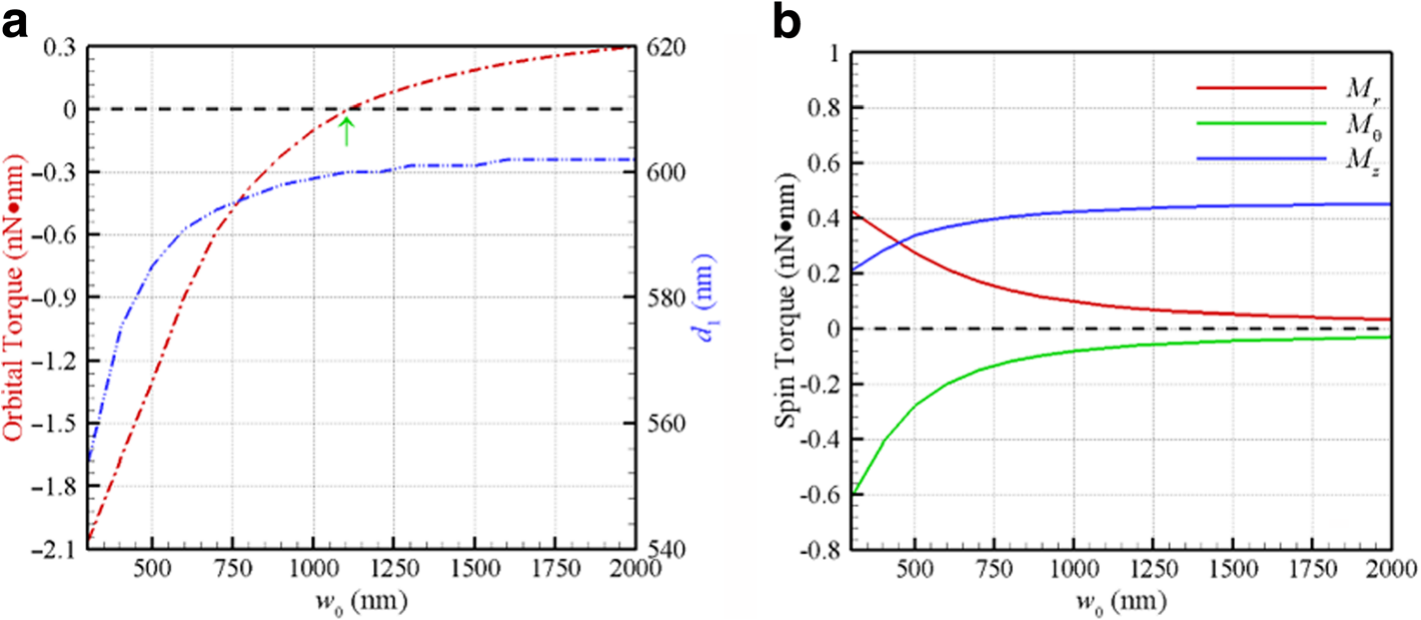
**a** The optical orbital torque and *d*_1_ at the first stable-equilibrium orbit versus the waist of a Gaussian beam of *λ* = 800 nm. The turning point of the waist for producing positive or negative orbital torque is at 1150 nm. **b** The optical spin torques versus the waist at *d*_1_

Furthermore, one can adjust the iris of object of optical tweezers to change the size of the incident beam, thereby altering the numerical aperture and the waist of a Gaussian beam. Figure 4a shows the optical orbital torque *F*_*θ*_ *d*/2 on two coupled Au NPs of radius 100 nm rotating at the corresponding first stable-equilibrium orbit (*d* = *d*_1_) versus the waist of a CP Gaussian beam of *λ* = 800 nm. The corresponding first stable-equilibrium distance *d*_1_ is also plotted in Fig. 4a (scale bar in the right side), where a plane wave can be treated as a special case of *w*_0_ → ∞. The turning point for the waist of a Gaussian beam between the positive and negative orbital torques is at 1150 nm, corresponding to *F*_*θ*_ = 0, as shown in Fig. 4a. The smaller the waist of a Gaussian beam is, the larger the magnitude of negative orbital torque. As the waist increases, the *d*_1_ of a Gaussian beam approaches the value (603 nm) of a plane wave (*w*_0_ → ∞). In particular, as the waist decreases, the magnitudes of the transverse spin torques (*M*_*r*_, *M*_*θ*_) at *d*_1_ significantly increase, while the longitudinal spin torque *M*_*z*_ decreases, as shown in Fig. 4b. These results illustrate that the waist of a Gaussian beam is the key factor to induce a negative longitudinal orbital torque and transverse spin torques due to the degree of the distortion of EM field.

The mechanism of the negative orbital rotation and the transverse spinning of these NPs could be attributed by the curl of the light field’s spin angular momentum, even without the contribution of the light beam’s orbital angular momentum [23]. Through the performances of the negative longitudinal orbital torque and the transverse spin torques upon Au dimer, the plasmon-enhanced SOC of photons can be manifested. In addition, the directions of the orbital rotation of the dimer and the spin of individual NP depend on the handedness of incident light.

## Conclusions

The optomechanical responses (optical forces and torques) of a pair of Au NPs irradiated by CP light were studied theoretically. Our results showed that the stable-equilibrium orbits for their rotation (revolution) can be induced for the long-range interaction; the stable-equilibrium interparticle distances are nearly the integer multiples of wavelength in medium. The azimuthal optical force causes the orbital rotation of these NPs, and the optical spin torque induces their spinning, particularly the transverse components. This motion is similar to that of binary stars of equal mass moving in a circular orbit around their common center of mass. When the waist of a Gaussian beam is smaller than a turning point, the helicity of the orbital rotation of the optically bound Au dimer is opposite to the handedness of the incident CP light. Moreover, the longitudinal/transverse SOC becomes significant as the waist of a Gaussian beam decreases; therefore, the transverse spin of the two NPs becomes more profound. For the short-range interaction, the optical force causes the mutual attraction. Consequently, the two coupled plasmonic NPs not only spin but also rotate with a spiral trajectory and will collide eventually. In addition, the borderline of the interparticle distance between the long-range and short-range interactions of two coupled NPs roughly is at a half wavelength in the medium. Our results demonstrated that the order of magnitude of optical force is about pN, which can be compared with the other forces (e.g., ponderomotive force) to identify the contribution on NPs’ motion. Our finding may pave the way to the applications of SOC on light-manipulating NPs for nanoscience and nanotechnology. Furthermore, it is worth studying the correlation between the optical spin and orbital torques on the two NPs and the spin and orbital angular momentum densities of EM field; the former is defined by $\operatorname{Im}\left(\overline{\mathbf{E}}\times \mathbf{E}\right)/2\omega$ and the latter shown in Ref. [23]. In addition, the SOC in the twisted near field of metamaterials is worth investigating [40–43].

## Appendix

The formulations of the electric field of LH CP plane wave and Gaussian beam with common time factor *e*^−*jωt*^ are given as follows, where *k* is the wavenumber in the surrounding medium.

1. Plane wave

$$ {E}_x=\frac{E_0}{\sqrt{2}}{e}^{jkz},{E}_y=\frac{jE_0}{\sqrt{2}}{e}^{jkz},{E}_z=0. $$

(2) Gaussian beam

$$ {E}_x\approx \frac{E_0{e}^{-{\rho}^2/\left(1+{\left(z/{z}_0\right)}^2\right)}}{\sqrt{2}\sqrt{1+{\left(z/{z}_0\right)}^2}}{e}^{j\left\{ kz\left[1+{z}_0{\rho}^2/\left({kz}^2+{kz}_0^2\right)\right]-{\tan}^{-1}\left(z/{z}_0\right)\right\}}, $$

$$ {E}_y\approx \frac{jE_0{e}^{-{\rho}^2/\left(1+{\left(z/{z}_0\right)}^2\right)}}{\sqrt{2}\sqrt{1+{\left(z/{z}_0\right)}^2}}{e}^{j\left\{ kz\left[1+{z}_0{\rho}^2/\left({kz}^2+{kz}_0^2\right)\right]-{\tan}^{-1}\left(z/{z}_0\right)\right\}}, $$

$$ {E}_z\approx \frac{-j\left({xE}_x+{yE}_y\right)}{z_0\sqrt{1+{\left(z/{z}_0\right)}^2}}{e}^{-j{\tan}^{-1}\left(z/{z}_0\right)}, $$

where $$ {z}_0={kw}_0^2/2 $$ and $$ \rho =\sqrt{\left({x}^2+{y}^2\right)/{w}_0^2} $$.

## Abbreviations

CP: Circularly polarized
EM: Electromagnetic
LH: Left-handed
LP: Linearly polarized
MMP: Multiple multipole
MP: Microparticle
NP: Nanoparticle
SOC: Spin-orbit coupling
